# Biodegradable Interpolycomplexes for Anti-Erosion Stabilization of Soil and Sand

**DOI:** 10.3390/polym14245383

**Published:** 2022-12-08

**Authors:** Olga A. Novoskoltseva, Andrey A. Belov, Nataliya G. Loiko, Yury A. Nikolaev, Irina G. Panova, Alexander A. Yaroslavov

**Affiliations:** 1 Faculty of Chemistry, Lomonosov Moscow State University, 119991 Moscow, Russia; 2 Faculty of Soil Science, Lomonosov Moscow State University, 119991 Moscow, Russia; 3 Department of Microbiology, Federal Research Center “Fundamentals of Biotechnology” RAS, 119071 Moscow, Russia

**Keywords:** alginate, quaternized hydroxyethyl cellulose ethoxylate, interpolyelectrolyte complex, soil, sand, erosion, stabilization, biodegradation

## Abstract

A linear anionic polysaccharide, sodium alginate, electrostatically interacts with a cationic polysaccharide, quaternized hydroxyethyl cellulose ethoxylate, in aqueous solution, thus giving an interpolyelectrolyte complex. Aqueous solutions of the initial polysaccharides and polycomplexes with an excess of the cationic or anionic polymers were used for the stabilization of soil and sand against water erosion. Physicochemical, mechanical and biological properties of the polymers and coatings were characterized by gravimetric analysis, viscosimetry, mechanical strength assessment, cell viability, and cell-mediated degradation with the following main conclusions. (a) Non-stoichiometric polycomplexes with an excess of cationic or anionic units (“cationic” and “anionic” polycomplexes, respectively) form transparent solutions or stable-in-time dispersions. (b) The complexation results in a decrease in the viscosity of polymer solutions. (c) A complete dissociation of polycomplexes to the initial components is achieved in a 0.2 M NaCl solution. (d) Soil/sand treatment with 1 wt% aqueous solutions of polymers or polycomplexes and further drying lead to the formation of strong composite coatings from polymer(s) and soil/sand particles. (e) Cationic polycomplexes form stronger coatings in comparison with anionic polycomplexes. (f) The polymer–soil coatings are stable towards re-watering, while the polymer–sand coatings show a much lower resistance to water. (g) The individual polysaccharides demonstrate a negligible toxicity to Gram-negative and Gram-positive bacteria and yeast. (h) The addition of *Bacillus subtilis* culture initiates the degradation of the polysaccharides and polycomplexes. (i) Films from polysaccharides and polycomplexes decompose down to small fragments after being in soil for 6 weeks. The results of the work are of importance for constructing water-resistant, low toxicity and biodegradable protective coatings for soil and sand.

## 1. Introduction

Soil is a unique natural resource, whose state is of exceptional importance not only for food production, but also for maintaining ecosystems on our planet [1,2]. The assessment of the current ameliorative state of soil in various countries indicates its pronounced degradation, which is expressed in a decrease in fertility, a loss of nutrients available to plants, contamination with toxic compounds, dehumidification and desertification [3,4,5,6]. In Russia, more than a third of agricultural soils are subjected to negative processes [7]. Although soil is considered a renewable resource, the development of soil lasts hundreds to thousands of years [8]. The degradation of soil primarily results from erosion, a destruction of the surface soil layer by water and wind [9]. Natural erosion is exacerbated by mechanical disturbance of the soil structure during intensive tillage, violation of the agricultural technology rules, and uncontrolled grazing [10,11].

Various approaches have been developed for enhancing the erosion resistance of soil [12,13], among which are chemical methods based on the use of synthetic and natural polymers [14]. Typically, an aqueous solution of polymer is deposited over the soil that, after drying, leads to the formation of a polymer–soil coating, of ca. 5 mm in thickness, which prevents the removal of soil with water and wind [13]. For many years, polyacrylamide and copolymers of acrylamide with anionic monomers have been taken for soil stabilization [15,16,17,18].

Recently, an alternative type of polymeric stabilizers has been suggested: interpolyelectrolyte complexes (IPEC), which are amphiphilic products of the electrostatic interaction between two oppositely charged ionic polymers (polyelectrolytes), one anionic and the other cationic [13,19,20,21]. IPECs are copolymers with sufficiently extended hydrophilic and hydrophobic blocks. They interact with complementary sites on the surface of soil particles and stick them together [13]. Variation of the IPEC composition allows adjusting its structure to a soil to be treated and controlling the mechanical strength and anti-erosion resistance of the resulting protective coatings [14,22]. In addition to the anti-erosion effect, polycomplex formulations can retain water in the soil, bind heavy metals and stimulate plant development [14]. 

Recently, polymers of natural origin, mainly polysaccharides, i.e., salts of alginic acid and carboxymethyl cellulose, chitosan, etc., have been put into practice [23,24,25,26,27]. Physical factors and soil microorganisms stimulate degradation of these biopolymers down to low molecular weight compounds, thus significantly reducing the environmental load on the soil. At the same time, the polysaccharide IPECs, described in the literature, are mostly stoichiometric formulations with an equimolar charge-to-charge ratio of cationic and anionic biopolymers [23,26,27]. Such IPECs are insoluble in water that makes them difficult to work with [13,26]. 

In the current work, we describe the interaction between oppositely charged polysaccharides, anionic sodium alginate (ALG) and cationic quaternized hydroxyethyl cellulose ethoxylate (QHECE), which allowed the non-stoichiometric biopolymer complexes to show long-term stability against aggregation in aqueous solutions due to an abundant charge of either polymer component. By varying the composition of the polycomplex, the mechanical properties of the protective coatings over sand and sandy soil were optimized. Special attention was paid to the toxicity of biopolymer formulations and their destruction induced by soil microorganisms. The data are relevant for the constriction and practical application of environmentally friendly biopolymer-based IPECs as soil conditioners.

## 2. Materials and Methods

### 2.1. Materials

ALG and QHECE were purchased from Sigma-Aldrich Co. (St. Louis, MO, USA) and used as received. The viscosity of 1 wt% ALG aqueous solution with a pH of 6.6 was 15 cSt (at 22 °C) and the viscosity of 1 wt% QHECE aqueous solution with a pH of 5.2 was 25 cSt (at 22 °C). 2,3,5-Triphenyltetrazolium chloride (TTC), sodium hydroxide, sodium chloride, sodium tetraborate, sodium dihydrogen phosphate monohydrate (all purchased from Sigma-Aldrich Co., St. Louis, MO, USA), hydrochloric acid and acetone (both purchased from Chimmed, Moscow, Russia) were used as received. 

Molar concentrations of anionic ALG groups and cationic QHECE groups were determined via potentiometric and turbidimetric titration, as described in [22,28], respectively. 

A soil sample (taken in May 2020) was described in detail earlier [22]. Its granulometric composition was obtained using a Mastersizer 3000E laser particle sizer (Malvern Instruments Ltd., Malvern, UK); 83% of particles were of 0.1–1 mm sizes and 17% of particles had lower sizes.

Quartz sand with 0.1–0.2 mm particles (ORT6, Moscow, Russia) was repeatedly washed with bi-distilled water before use. 

The water was purified as described in [29] and showed a specific conductivity of 0.6 μS cm^–1^.

### 2.2. Methods

Optical densities of solutions were measured with a Genesys™ 50 UV–Visible spectrophotometer (Thermo Fisher Scientific, Madison, WI, USA) using quartz cuvettes with a width of 1 cm.

The pH measurements were performed using a Corning 340 pH meter (Corning Inc., Corning, NY, USA) equipped with a combination glass pH electrode with an integrated temperature sensor.

The kinematic viscosity of polymer solutions was measured immediately after the sample preparation and then every 5–6 days using capillary viscometer VPJ-2 0.99 and viscometer VPJ-2 1.77 (Ecroskhim Ltd., Saint-Petersburg, Russia). The choice of instrument was determined by the solution viscosity. Experimentally, the expiration time for the polymer solution was determined, which was recalculated to the kinematic viscosity (*η*) according to (1):

(1) $$\eta =\frac{gtK}{9.807}$$

where *g* is the acceleration of gravity at a specific point of measurements, *t* is the expiration time, and *K* is the viscometer constant equal to 1 for a 1.77 mm capillary and 0.1 for a 0.99 mm capillary.

The mechanical strength of protective polymer–soil and polymer–sand crusts was tested following the procedure described in [30]. Briefly, a soil (or sand) sample was placed in a Petri dish covered with a 1 wt% polymer aqueous solution and dried, resulting in a polymer–soil (sand) crust on the top. The mechanical properties of the crusts were studied with a Rebinder conical plastometer (Faculty of Soil Science, Lomonosov Moscow State University) [30] and presented as the maximum pressure before sample destruction (strength of the crust, *P_m_*).

Anti-erosion experiments were performed according to the protocol described in [31]. Soil (or sand) in a Petri dish with a deposited aqueous polymer solution was dried; the dish was turned to an angle of 45° and sprayed with water. After drying, the weight loss was calculated.

In the antimicrobial experiments [22], three types of microorganisms were used: Gram-negative bacteria *Pseudomonas aeruginosa* 4.8.1, Gram-positive bacteria *Staphylococcus aureus* 209P and yeast (eukaryotes) *Yarrowia lipolytica* 367–2 (microorganism collections of the Research Center of Biotechnology RAS, Moscow, Russia). The lowest polymer concentration which did not yet inhibit the growth of the test cultures was taken as the minimum inhibitory concentration (MIC). The lowest polymer concentration which did not inhibit the microorganism growth on agar media was taken as the minimum bactericidal concentration (MBC) [32,33,34,35]. 

To study the biodegradation of polysaccharide formulations, polypropylene flaks with 100 mL of 1 wt% colorless polymer solutions or 1 wt% white polycomplex dispersions in a 10^−2^ M phosphate buffer of pH 7 were sterilized for 30 min at 112 °C. Then, 50 μL of *Bacillus subtilis* cells (VKM B-501), grown in Evans medium and pre-washed in a sterile pH 7 10^−2^ M phosphate buffer, was added to each flask at a final concentration of 0.1 g L^−1^ (final population density was 10^2^ CFU mL^−1^). TTC was added to the flasks as an indicator of microbial dehydrogenase activity [36,37], and the flaks were incubated for a month under aerobic conditions at 28 °C in a Memmert 100–800 air-dry oven (Memmert GmbH & Co. KG, Schwabach, Germany). A change in the color of a polymer solution/suspension to rose-red indicated the bacteria-induced degradation of polysaccharides.

Films for biodegradation testing were prepared as follows: 5 mL of 1 wt% polysaccharide solution or 1 wt% IPEC solution was deposited over a 10 cm^2^ polypropylene substrate and dried at room temperature for 2 days to constant weight. The dried films, typically 30 µm in thickness, were removed from the substrate and their biodegradation was analyzed following the soil burial method [38,39,40]. Films of 4 cm^2^ area were placed into cups made of a polyester mesh to permit the access of microorganisms and moisture and to easily take out the degraded films. The cups were buried in 100 g of the soil located in round polypropylene flasks (12.5 cm^2^ × 12 cm) at a depth of 6 cm in order to ensure aerobic degradation conditions. The procedure was performed at 28 °C and 35% water content of the soil by adding water periodically. After 6 weeks, the polyester cups were taken out, and the samples were washed carefully with water. Biodegradation was assessed qualitatively from photographs.

## 3. Results and Discussion

### 3.1. Formation and Properties of ALG–QHECE Polycomplexes

**Figure 1:**
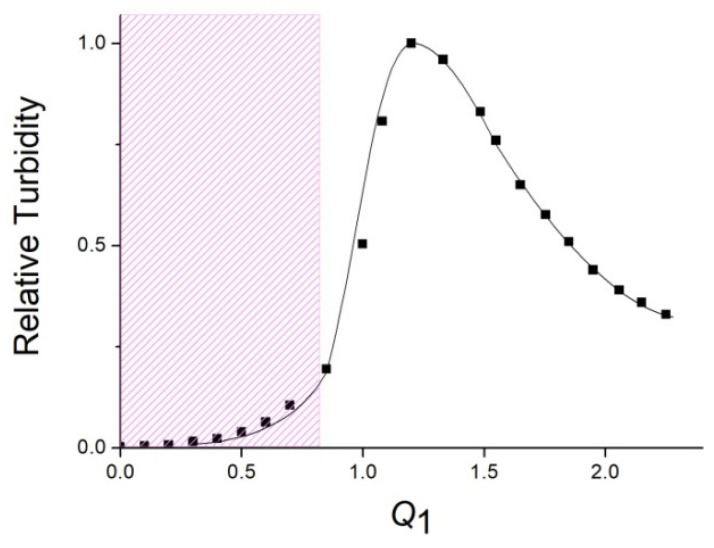
Relative turbidity of IPEC solution at *λ* = 500 nm vs. *Q*_1_ = [ALG]/[QHECE]. [QHECE] = 5 × 10^−4^ M; 10^−2^ M phosphate buffer of pH 7. The cross-hatched region represents water-soluble IPECs.

An ALG-to-QHECE electrostatic complexation was studied in a 10^–2^ M phosphate buffer solution of pH 7. Upon the addition of an ALG solution and to a QHECE solution, the resulting mixture remained transparent up to a molar ratio *Q*_1_ = [ALG]/[QHECE] = 0.8 (Figure 1). In this *Q*_1_ value range, water-soluble interpolyelectrolyte complexes (IPEC) were formed with an excess of cationic QHECE groups. At *Q*_1_ > 0.8, the mixtures became progressively turbid due to the formation of insoluble IPEC.

The cationic QHECE contains quaternized amino groups which give the maximum positive charge to a QHECE macromolecule at pH 7 where the complexation was examined. In other words, all the cationic QHECE groups participated in the electrostatic bonding to ALG. As shown earlier, the maximum turbidity, or the maximum size of particles, in the polyanion–polycation binary system is observed at an equimolar charge-to-charge ratio of both polymer components [29,41]. It follows from there that the maximum turbidity is reached when the concentration of the complexed anionic (ionized) ALG groups is equal to the concentration of the cationic QHECE groups: [ALG^−^]/[QHECE] = 1, or at *Q*_1_ = [ALG]/[QHECE] = 1.2 (Figure 1). This allows the estimation of the maximum degree of anionic ALG groups involved in the electrostatic complexation with QHECE: *ω* = [ALG^−^]/[ALG] = 1/1.2 = 0.83. Residual 1−0.83 = 0.17 anionic ALG groups did not interact with cationic QHECE. The *ω* = 0.83 and *Q*_1_ = 1.2, thus, corresponded to the electroneutral (saturated) ALG–QHECE polycomplex at pH 7. The further increase of *Q*_1_ over 1.2 resulted in a decrease in the turbidity that reflected the formation of smaller IPEC particles stabilized by the negative charge of ALG taken in an excess. Water-soluble IPECs can be regarded as block-copolymers with hydrophobic fragments from mutually neutralized cationic and anionic groups of both polymers and the charged units of polymer taken in excess [19,20]. 

**Figure 2:**
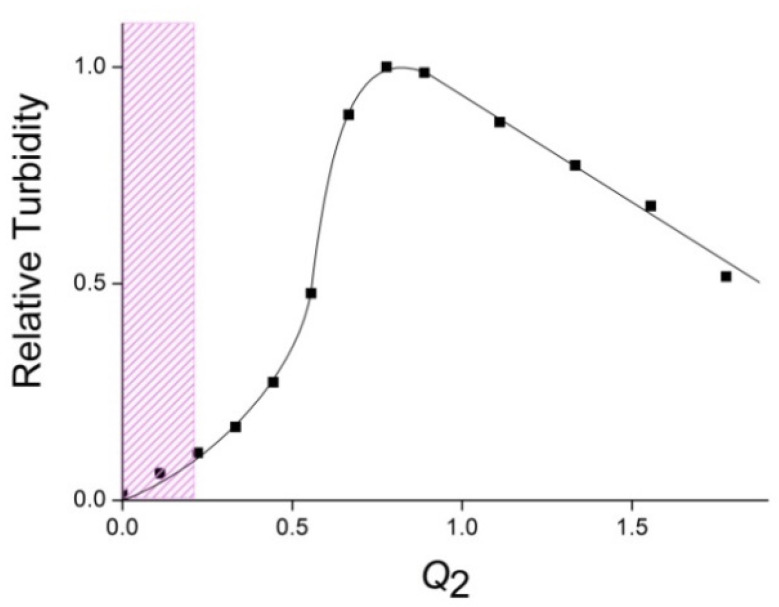
Relative turbidity of IPEC solutions at *λ* = 500 nm vs. *Q*_2_ = [QHECE]/[ALG]. [ALG] = 10^–3^ M; 10^–2^ M phosphate buffer of pH 7. The cross-hatched region represents water-soluble IPECs.

When a QHECE solution was added to an ALG solution, i.e., at a “reverse order” of polymer mixing, the saturated IPEC with maximum turbidity was achieved at *Q*_2_ = [QHECE]/[ALG] = 0.8 (Figure 2), while *Q*_2_ = 1/*Q*_1_. The data of both figures show that the order of polymer mixing gave saturated IPECs of the same composition. It should be noted, however, that unsaturated (non-stoichiometric) IPECs differs in terms of their stability against aggregation. IPECs with an excess of the cationic QHECE (“cationic IPECs”) gave transparent solutions until *Q*_1_ = 0.8 (Figure 1), while IPECs with an excess of the anionic ALG (“anionic IPECs”) gave transparent solutions only until *Q*_2_ = 0.2 (Figure 2). In other words, water-soluble cationic IPECs may contain more mutually neutralized (hydrophobic) fragments in comparison with water-soluble anionic IPECs. The reason for such behavior of cationic and anionic IPECs is not yet clear. It could be connected, for instance, with a different affinity of small counter-ions to cationic QHECE and anionic ALG chains [19], or different molecular masses of these polymers, as indicated by varying viscosities of their aqueous solutions.

**Figure 3:**
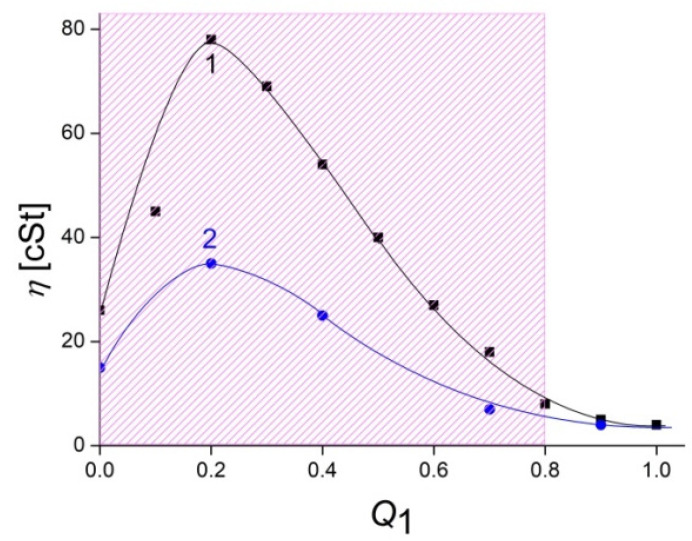
Kinematic viscosity (*η*) of IPEC solutions vs. *Q*_1_ = [ALG]/[QHECE]. [QHECE] = 1.5 × 10^–2^ M; 10^–2^ M phosphate buffer of (**1**) pH 7 and (**2**) pH 10. The cross-hatched region represents water-soluble IPECs.

IPEC formation is accompanied by mutual neutralization of polyelectrolyte charges that results in a decrease of the size of macromolecular coils [41] and a progressive lowering of the viscosity of solutions [42]. However, contrary to expectations, the “viscosity vs. *Q*_1_ = [ALG]/[QHECE]” plot for the cationic IPECs in the buffer solution of pH 7 is described by a curve with a pronounced maximum at *Q*_1_ = 0.2 (Figure 3, curve 1), but not a smooth falling curve. The increase in viscosity can be explained as follows. The viscosity of an initial 1 wt% solution of QHECE is rather high, i.e., 25 cSt, which obviously reflects a high molecular mass of the polymer and its large total charge due to quaternized amino groups. These parameters determine an extended conformation of QHECE macromolecules in aqueous solution. When the first aliquots of ALG solution are added to the 1 wt% QHECE solution, ALG macromolecules interact with QHECE macromolecules, thus forming a network in which each macromolecule electrostatically binds to several oppositely charged macromolecules [43,44,45]. Additionally, non-ionized ionic groups of ALG can be involved in the intermolecular H-bond formation that also contributes to the network stabilization [46]. This leads to an increase in the viscosity of the system, and the formation of a gel-like IPEC structure in the bulk [19]. A further increase in the ALG concentration results in a progressive neutralization of the QHECE charges that is accompanied by a decrease in the amount of intermolecular contacts, destroying the network and lowering the viscosity, which returns the viscosity vs. *Q*_1_ curve to its usual falling form.

The significant role of H-bonds in the stabilization of the cationic ALG–QHECE network at a lower ALG content was demonstrated during the following experiment. The ALG-to-QHECE complexation was carried out in a solution of pH 10 (Figure 3, curve 2), where nearly all ionic groups of ALG were ionized. Under these conditions, the maximum viscosity of IPEC solutions was halved compared to the viscosity measured in a solution of pH 7 (cf. curves 1 and 2 in Figure 3). 

**Figure 4:**
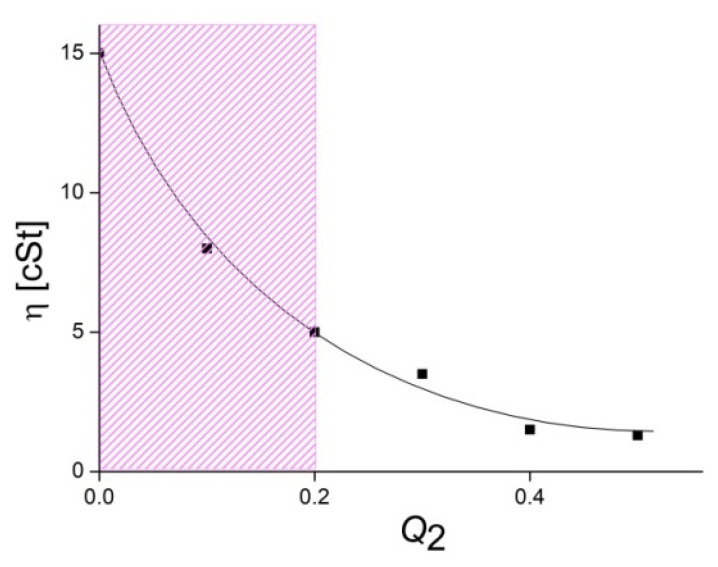
Kinematic viscosity of IPEC solutions vs. *Q*_2_ = [QHECE]/[ALG]. [ALG] = 5 × 10^−2^ M; 10^−2^ M phosphate buffer of pH 7. The cross-hatched region represents water-soluble IPECs.

A reverse viscosity titration, when a QHECE solution was added to an ALG solution, gave a “viscosity vs. *Q*_2_ = [QHECE]/[ALG]” plot for the anionic IPECs, which was described with a smooth falling curve, as shown in Figure 4. In this case, QHECE aliquots were introduced in a 1 wt% solution of ALG with a lower initial viscosity: 15 cSt against 25 cSt for the 1 wt% QHECE solution. The lower viscosity was obviously associated with a lower molecular mass of ALG that did not allow the formation of a polymer network after the addition of QHECE and an increase in the viscosity of the mixed ALG–QHECE solution.

**Figure 5:**
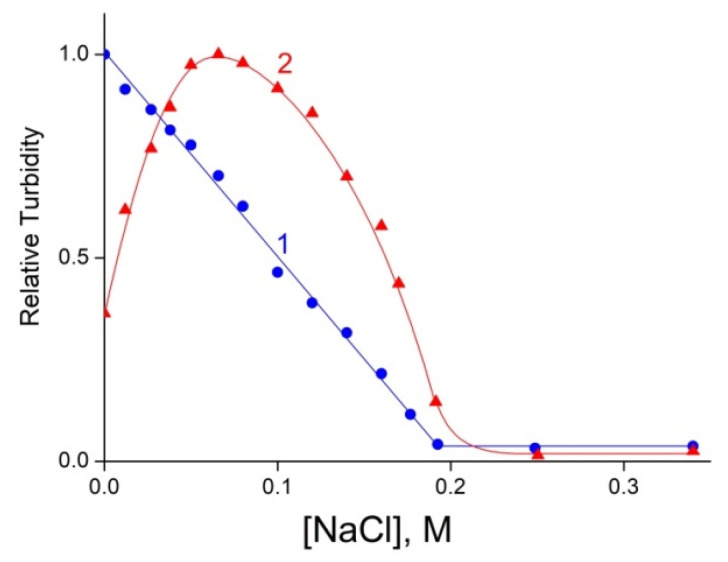
Relative turbidity of IPEC solutions at *λ* = 500 nm vs. NaCl concentration. (**1**) Water-soluble cationic IPEC with *Q*_1_ = [ALG]/[QHECE] = 0.5, [QHECE] = 5 × 10^−3^ M and (**2**) water-soluble anionic IPEC with *Q*_2_ = [QHECE]/[ALG] = 0.2, [ALG] = 5 × 10^−3^ M; 10^−2^ M phosphate buffer of pH 7.

The electrostatic IPECs, stabilized by multiple salt bonds between anionic and cationic groups of polymers, are sensitive to the concentration of salt in solution [19,20]. This scheme manifested itself differently for cationic and anionic IPECs. An increase in NaCl concentration in a cationic IPEC solution (curve 1 in Figure 5) led to a gradual decrease in the turbidity and, finally, to a transparent solution at a 0.2 M NaCl concentration, which reflected a successive dissociation of the network-arranged IPEC down to the individual components, ALG and QHECE. As for the anionic IPEC solution (curve 2 in Figure 5), the addition of salt first caused an increase in the turbidity, which then decreased and disappeared in a 0.2 M NaCl solution, as in the case of the cationic IPEC. A rise in the turbidity showed the formation of stoichiometric IPEC due to intra- and intermolecular ion-exchange reactions induced by the addition of salt in conventional IPEC solutions [20]. Such reactions were either prohibited in the cross-linked cationic IPEC or proceeded at a very slow rate.

### 3.2. Polysaccharides and Polycomplexes for the Protection of Soil and Sand against Water Erosion

**Figure 6:**
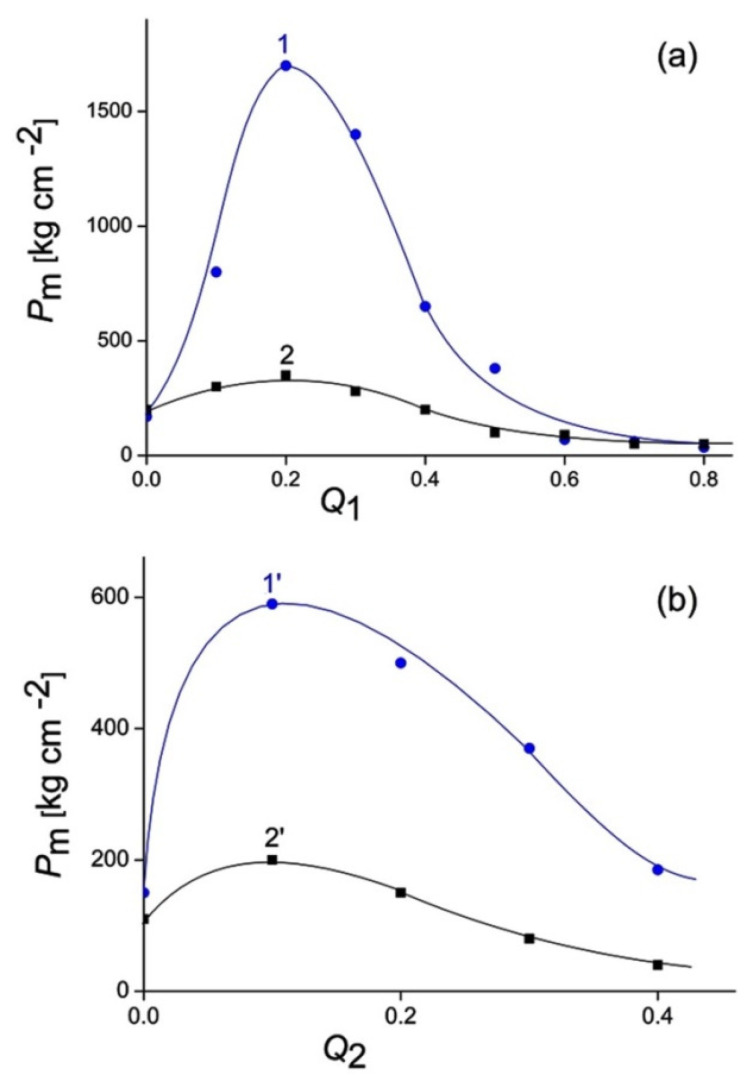
Mechanical strength of polymer–soil/sand crusts vs. composition of deposited IPEC formulations. (**a**) Cationic IPEC and (**b**) anionic IPEC; sand (1 and 1′) and soil (2 and 2′).

Sandy soil, which contained 83% of 0.1–1 mm particles, and quartz sand, whose grain size was 0.1–0.2 mm, were used as substrates for the preparation of protective coatings. As such, 1 wt% polymer formulations, aqueous solutions of cationic and anionic IPECs, were deposited over the soil/sand substrate surface; aqueous solutions of the individual polymers, ALG and QHECE, were taken as a control. The deposition of the polymer formulations and further drying of the samples led to the formation of polymer–soil/sand crusts. The mechanical strength of the crusts, hereinafter referred to as *P*_m_, is shown in Figure 6 as a function of the composition of the deposited IPECs. Three points in the figure deserve special attention. First, the cationic IPECs form stronger crusts in comparison with the anionic (sf. a and b). This is most likely due to the electrostatic binding of free cationic moieties of IPECs to anionic sites on the surface of quartz particles, and mixtures of various soil particles containing sand/clay/humic acids. Second, the “*P*_m_ vs. *Q*_1_” and “*P*_m_ vs. *Q*_2_” plots have maxima, less pronounced for soil and more pronounced for sand, at *Q*_1_ = *Q*_2_ = 0.1–0.2 (cf. in pair curves 1 and 2 and curves 1’and 2’). Thus, the crusts with the greatest strength are obtained with the use of IPECs, in which only a small part of polymer, taken in an excess, is involved in IPEC formation. This may indicate that charge-to-charge interactions are the main contributors to soil stabilization. Third, the IPECs with a high content of mutually neutralized charges (with high *Q*_1_ and *Q*_2_ values) provide the crusts with mechanical strength that is comparable to or even lower than the strength of crusts from the individual polymers (cf. left and right parts of each curve in Figure 6). This fact is in accordance with the above statement about a decisive contribution of electrostatic forces in the binding of ALG–QHECE IPECs with soil and sand particles. In general, the strength of the sand-based crusts turned out to be higher than the soil-based crusts, obviously due to the higher strength of sand particles, which is the main component of the resulting crust. 

As shown earlier [22], 1 wt% aqueous polymer formulations penetrate into the soil and sand to a depth of 5 mm at a consumption rate of 2.5 L/m^2^ for soil and 1.5 L/m^2^ for sand. In the current article, 1 wt% aqueous formulations of anionic and cationic polysaccharides and their polycomplexes were deposited over soil and sand using the same protocol. As expected, an average thickness of polymer–soil/sand crusts turned out to be close to the predicted value: from 4 up to 7 mm.

The samples with protective crusts were tested for resistance to water erosion. In control experiments, the initial soil/sand was placed in a Petri dish and treated with 200 mL of water within 10 min, accompanied by a 55–60% washout of both substrates. The crusts formed by the individual polysaccharides and soluble IPECs demonstrated different results depending on what type of substrate was treated (Table 1). The soil was effectively stabilized by the individual polymers with a soil washout of less than 5%, and especially by IPECs, both cationic and anionic, with a washout of less than 1% (columns 2 and 3 in Table 1). Contrastingly, stabilization of the sand by the same formulations was much weaker, while the sand treated by cationic QHECE and cationic IPECs showed the lowest resistance to water (columns 4 and 5 in Table 1). The reason for the better water resistance of the polymer–soil crusts compared to the polymer–sand crusts is not yet clear. It is likely due to the small < 0.05 mm particles (physical clay and dust) and organic matter with a total content of ca. 15% in the soil sample [47]. The small particles decreased the porosity of soil, thus hindering the penetration of water into the crust and polymer washout. The organic matter was able to form non-ionic contacts with polymers with a lower sensitivity to water. Complexation of QHECE with ALG was accompanied by the formation of hydrophobic blocks from mutually neutralized units of both polymers and additional stabilization of the resulting crusts to water. 

### 3.3. Biological Activity of Polysaccharide Formulations

The results of assessing MIC and MBC values for the initial polysaccharides, QHECE and ALG, are summarized in Table 2. Both polymers did not suppress the growth of all three tested microorganisms even at the highest tested polymer concentration of 1 wt%. In other words, the MICs and MBCs exceeded 1 wt% concentration that is reflected in Table 2 by the “>1” symbol. If the anionic ALG showed negligible toxicity as expected, then a cationic QHECE’s low toxicity was a pleasant surprise. We see in this example that the attachment of potentially toxic cationic amino groups to a biodegradable polysaccharide may not lead to toxicity in the final polymeric product. This observation is useful for designing polymers (polymeric complexes, conjugates, etc.) contacting with biological media.

**Figure 7:**
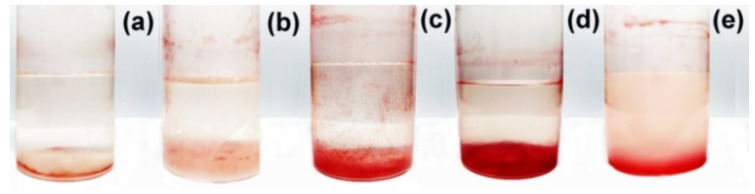
Images of the polysaccharide formulations after 1 month of incubation with *B. subtilis* in the presence of TTC at 28 °C: (**a**) ALG; (**b**) dispersion of anionic complexes with *Q*_2_ = [QHECE]/[ALG] = 0.1; (**c**) QHECE; (**d**) dispersion of cationic complexes with *Q*_1_ = [ALG]/[QHECE] = 0.2; (**e**) dispersion of cationic complexes with Q_1_ = [ALG]/[QHECE] = 0.6.

Biodegradation of the polysaccharide formulations was initiated by the addition of a *B. subtilis* culture to the polymer solutions/dispersions in the pH 7 phosphate buffer. TTC was additionally added as an indicator of microbial dehydrogenase activity. After a month of incubation at 28 °C, the solutions/dispersions became rose-red (Figure 7), definitely indicating a biodegradation process, which was induced by dehydrogenase enzymes of *B. subtilis* bacteria, and preservation of the cell viability. It should be noted that the bacteria ate anionic and cationic polysaccharides and polycomplexes equally well, thereby confirming the above conclusion about the non-toxicity of all prepared polymer formulations.

**Figure 8:**
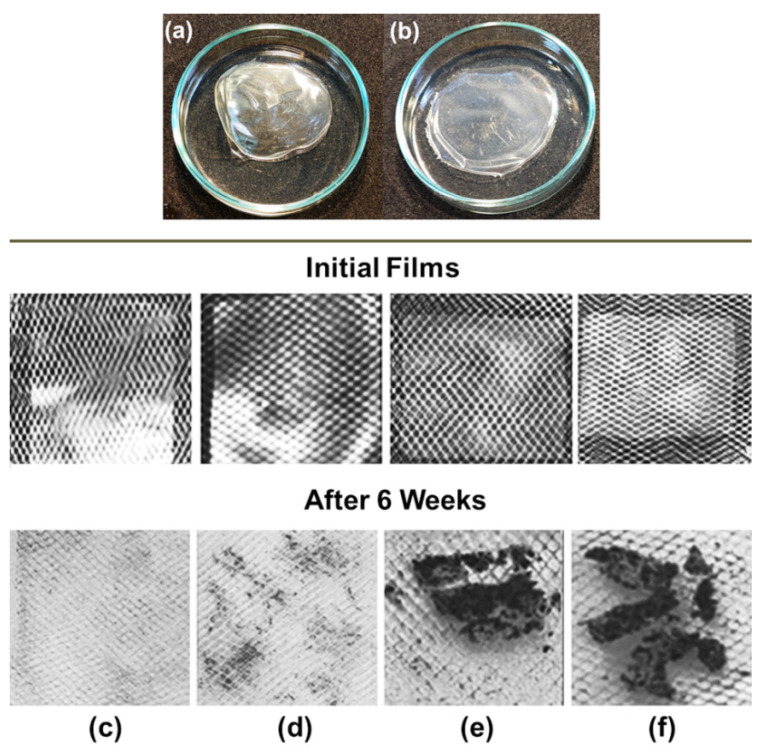
Images of films from (**a**) ALG and (**b**) anionic IPEC with *Q*_2_ = [QHECE]/[ALG] = 0.1. Cups with the initial film samples and the samples taken out of the cups after 6 weeks of being buried in soil: (**c**) ALG, (**d**) QHECE, (**e**) anionic complex with *Q*_2_ = [QHECE]/[ALG] = 0.1, (**f**) cationic complex with *Q*_1_ = [ALG]/[QHECE] = 0.2.

Finally, biodegradation of the films from the individual polysaccharides and IPECs was investigated. Figure 8 illustrates the initial films of ALG (a) and anionic IPEC with *Q*_2_ = [QHECE]/[ALG] = 0.1 (b). In soil, there is a wide diversity of microorganisms that produce various enzymes involved in biodegradation processes [48]. Degradation of the polymer films in soil was controlled visually. Figure 8 shows photos of the films after being kept in soil for 6 weeks. The film from individual ALG was completely destroyed (c), and the film from QHECE gave a few small fragments (d). On the contrary, two films from ALG–QHECE polycomplexes, anionic (e) and cationic (f), gave bigger fragments after 6 week of being kept in the soil. Thus, the stability of the polymer films in the soil increases in the order: ALG ˂ QHECE ˂˂ anionic polycomplexes ≈ cationic polycomplex.

## 4. Conclusions

A linear anionic polysaccharide, ALG, electrostatically interacts with a cationic polysaccharide, QHECE, in aqueous solutions, thus giving an interpolyelectrolyte complex. Non-stoichiometric anionic and cationic IPECs form transparent solutions or stable dispersions. The ALG-to-QHECE complexation results in a decrease in the viscosity of the polymer solutions. The IPECs dissociate in water–salt solutions; a complete dissociation down to the initial components is achieved in a 0.2 M NaCl solution. 

The deposition of 1 wt% aqueous solutions of polymers or IPECs over soil and sand and further drying leads to the formation of the composite coatings from polymer(s) and soil/sand particles. The cationic IPECs form stronger coatings in comparison with the anionic IPECs. The polymer–soil coatings are stable towards re-watering, whereas the polymer–sand coatings show a much lower resistance to water. 

The individual polysaccharides demonstrate negligible toxicity to Gram-negative and Gram-positive bacteria and yeast in aqueous solutions. The addition of *Bacillus subtilis* culture initiates the degradation of the polysaccharides and IPECs in solutions. Films from polysaccharides and IPECs decompose down to small fragments after being kept in soil for 6 weeks.

Based on the above results, water-resistant, low toxicity and biodegradable protective coatings for soil stabilization can be constructed.

## Figures and Tables

**Table 1:** Resistance of polymer–soil/sand crusts to water erosion.

1	2	3	4	5
Formulation	Loss of Soil, %	Crusts after Water Treatment	Loss of Sand, %	Crusts after Water Treatment
QHECE	<5	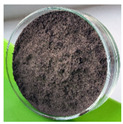	50	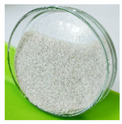
*Q*_1_ = 0.4	<1	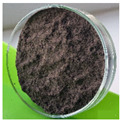	50	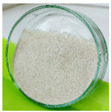
*Q*_1_ = 0.8	<1	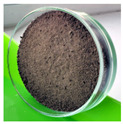	50	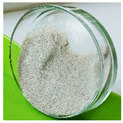
ALG	<5	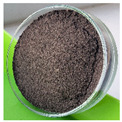	50	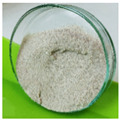
*Q*_2_ = 0.2	<1	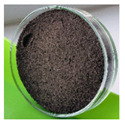	40	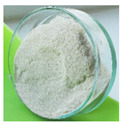
*Q*_2_ = 0.3	<1	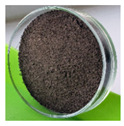	20	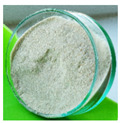
*Q*_2_ = 0.4	<1	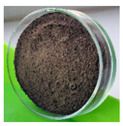	<10	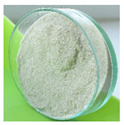

**Table 2:** Antimicrobial activity of polymer formulations in solution.

Formulation	Parameter	MIC and MBC, wt%		
		*P. aeruginosa*	*S. aureus*	*Y. lipolytica*
QHECE	MIC	>1	>1	>1
	MBC	>1	>1	>1
ALG	MIC	>1	>1	>1
	MBC	>1	>1	>1

## Data Availability

Not applicable.
